# Association between Physical Activity and Sleep Difficulties among Adolescents in Germany: The Role of Socioeconomic Status

**DOI:** 10.3390/ijerph18189664

**Published:** 2021-09-14

**Authors:** Miriam Blume, Petra Rattay

**Affiliations:** Department of Epidemiology and Health Monitoring, Robert Koch-Institute, 13353 Berlin, Germany; RattayP@rki.de

**Keywords:** sleep, physical activity, socioeconomic status, adolescents, health inequality, German Health Interview and Examination Survey for Children and Adolescents (KiGGS)

## Abstract

We examined sleep difficulties among adolescents in Germany and the association with physical activity (PA). Furthermore, we analyzed whether the association varied with the socioeconomic status (SES) among adolescent girls and boys in Germany. Using data from the German Health Interview Examination Survey for Children and Adolescents (KiGGS) study (Wave 2), 6599 adolescents aged 11 to 17 years were included in the analyses. We conducted sex-stratified logistic regression analyses. Dependent variables were unrecommended sleep duration (defined as a duration of sleep that does not meet the recommended duration), sleep-onset difficulties, trouble sleeping, and daytime sleepiness. Most adolescent girls and boys reported sleep difficulties. While no associations between PA and sleep difficulties were observed, a significant interaction between PA and SES was found for sleep duration in boys and daytime sleepiness in girls. Thus, adolescents with low SES had fewer sleep difficulties if they met the recommendation for PA, compared with those in other SES groups. In Germany, a large proportion of adolescents have sleep difficulties. We found that the experience of sleep difficulties varied according to PA, sex, and the family SES. Future sleep promotion programs should consider these differences.

## 1. Introduction

Sleep is highly important for the overall health and well-being of adolescents [1]. Sleep has been proven in several studies to be important for a variety of health outcomes [2,3,4]. Especially with respect to adolescents, extensive development in the body and brain can result in altered sleeping patterns during adolescence [1]. With the onset of adolescence, sleep behavior and sleep architecture change. Even if adolescents had good sleep quality as a child, this can change during adolescence [1]. 

Sleep covers various aspects and has many definitions. Sleep can be measured using different variables such as sleep duration, sleep problems, sleep quality, terminal wakefulness, sleep efficiency, and others [5,6,7,8,9]. Most studies use sleep duration in investigations of sleep among adolescents. Other sleep variables have not been well investigated [5,6]. The use of different indicators and definitions of sleep makes it difficult to compare study results [10]. The official recommendation for sleep duration of the American Academy of Sleep Medicine states that children from 6 to 12 years of age should sleep 9 to 12 h per 24 h on a regular basis to promote optimal health. Teenagers between 13 and 18 years of age should sleep 8 to 10 h per 24 h on a regular basis [11]. 

Looking at different sleep variables, studies have shown that between 47.8% and 65.5% of all adolescents meet the recommendation for sleep duration of a minimum 8 h of sleep per night [12,13,14]. This indicates that a large number of adolescents are not meeting the recommendation. The Health Behavior in School-aged Children (HBSC) study in Europe found that approximately 20% of children and adolescents reported sleep-onset difficulties [15]. Sleep-onset difficulties among European adolescents increased over time between 2002 and 2014, with slight variation among countries [15]. Trouble sleeping has been reported by 30.4% of Finnish children [16], and daytime sleepiness is present in 18% of Norwegian adolescents [5]. Data of adolescents in Germany are lacking regarding sleep-onset difficulties, trouble sleeping, and daytime sleepiness. 

Several systematic reviews focusing on children and adolescents have explored sleep and its association with other variables [3,4,17,18]. One systematic review found associations between sleep and general health outcomes, overweight, obesity, health conditions, school performance, and risk behaviors [4]. Focusing on sleep duration, the recommendations indicate that adverse health outcomes can occur in adolescents with short sleep durations. A significant association was found between short sleep duration and obesity or overweight [19] as well as declining self-rated health and health complaints [13]. In comparison, sufficient sleep has been shown to play an important role in the well-being of adolescents [14]. The reasons for insufficient sleep were investigated in a qualitative study in which adolescents mostly reported that insufficient sleep was owing to high academic and social requirements (e.g., school, activities, social environment) and their inability to cope with these because of personal circumstances. The main reasons for sleep difficulties and insufficient sleep among adolescents are stress, media use, and poor sleeping habits [20]. In addition to sleep duration, sleep-onset difficulty is another important sleep variable. Adolescents reporting excessive screen time were shown to have higher odds of reporting sleep-onset difficulties, which increased over time between 2002 and 2014 [15]. Sleep-onset difficulties are associated with perceived health status, life satisfaction, quality of family relationships, academic performance, and health complaints [8]. Sleep problems is another sleep indicator. One study reported a 16-fold increased risk of any psychosocial symptoms or somatic complaints among adolescents with sleep problems in comparison with adolescents without sleep problems. The same study also found an association between sleep problems and attention problems [16]. A review showed the association of morning tiredness and perceived health status, life satisfaction, quality of family relationships, academic performances, and health complaints. In that review, the associations between morning tiredness and health variables were higher than those between sleep duration and health [8].

An important variable interacting with sleep is physical activity (PA). Notably, the relationships between PA and sleep duration, sleep-onset difficulties, trouble sleeping, or daytime sleepiness have not often been analyzed [10,21,22]. PA is defined as “any bodily movement produced by skeletal muscles that results in energy expenditure” [23] (p. 126) and can be categorized as being of light, moderate, or heavy intensity. These categories are on the basis of the caloric expenditure of PA [23]. The World Health Organization (WHO) recommends children and adolescents to be physically active for a minimum of 60 min with moderate to heavy intensity every day. Being more physically active will lead to greater health benefits. The recommended daily amount of PA can serve as the minimum target for children and adolescents to achieve health benefits [24]. A clear association between PA and sleep has been described in a narrative review by Brand et al. [2]. Other studies have found good sleep quality and quantity among adolescents to be associated with an appropriate amount of PA [12] and that higher PA levels are associated with better quality sleep among adolescents in general [10,21]. One study found a causal relationship between PA and sleep quality. After exercising for 3 weeks, adolescents had better sleep quality compared with the control group [22]. Contrarily, a longitudinal study found that short or long sleep duration did not have an effect on the ability to perform moderate to vigorous PA 6 years later from the age of 9 to 15 [25].

In addition to PA, socioeconomic status (SES) is a critical factor in adolescents’ health. SES is known to be associated with several health outcomes and behaviors among adolescents [17]. Several reviews have shown a causal association between SES and PA [26,27,28]. Specifically, adolescents with a higher SES meet the WHO recommendation for PA more often than adolescents from families with a low SES, a fact that has not changed over the past several decades [29]. Furthermore, studies have found correlations between SES and sleep [30,31]. More specifically, a study has shown that low SES is associated with unfavorable sleep [30]. However, one study did not find a significant association of SES with sleep problems in children [17]. 

Other variables should be considered while investigating sleep. Sex has been found to play an important role in the context of sleep among adolescents [6,7,12,15,32,33,34]. Studies have reported mixed results. However, in girls and boys, different sleep habits, sleep durations, as well as sleep needs were observed [32,33,34]. Adolescents’ age is also a relevant factor in sleep problems [16] and sleep-onset difficulties [15], which increase with age. Additionally, screen time strongly influences the quality and quantity of sleep. Xu et al. [12] found that having fewer than 2 h of screen time is associated with good sleep. Besides the association between screen time and sleep, there were also an association between high screen time and low PA [25]. Additionally, studies have shown an association between sleep and mental health outcomes [3,5,14] as well as between sleep and personal resources [35,36], showing better resources and mental health outcomes were linked with better sleep quantity or quality. 

Current investigations are often lacking regarding the full complexity of sleep in adolescents, especially in Germany. Several studies have reported the importance of sleep for adolescents’ health [1,2,3,4]. However, only a few studies have focused on the association between sleep and PA. To our knowledge, no study to date has examined the moderating effects of family SES. Therefore, the aim of this paper was to analyze the relationship between PA and sleep difficulties among female and male adolescents as well as variations in the associations by SES. In detail, this paper addresses the following research questions:

- How many adolescent girls and boys in Germany have sleep difficulties (unrecommended sleep duration, sleep-onset difficulties, trouble sleeping, daytime sleepiness)?
- Are there differences in sleep difficulties according to PA between adolescent girls and boys?
- Do the associations between sleep difficulties and PA persist after adjusting for age, screen time, and personal resources, as well as emotional and behavioral problems?
- Does SES moderate the association between PA and sleep difficulties in adolescent girls and boys?

## 2. Materials and Methods

### 2.1. Data

The analyses in this study were conducted using data from the Health Interview and Examination Survey for Children and Adolescents (KiGGS) study. The KiGGS Wave 2 survey was carried out by the Robert Koch Institute from 2014 to 2017 as an interview and examination survey. Children and adolescents between 0 and 17 years of age, as well as their parents, participated voluntarily. Participants were chosen from the register of residents’ registration office, stratified by age. The KiGGS Wave 2 survey received ethics approval from the Hannover Medical School (Nr. 2275-2014). Further details about the study design have been published by Mauz et al. [37]. 

The data used in this study were from a sample of the cross-sectional study, with answers on the questionnaires given by the adolescents themselves. Only the information regarding SES was provided by the parents. In the present study, 6599 adolescents aged 11 to 17 years were included. The study population’s mean age was 13.84 (±1.98) years. In the sample, 51.87% were girls. The sample description is given in Table 1. 

### 2.2. Variables

In the present analyses, we used the definition and operationalization of sleep difficulties of Jakobsson et al. [20]. With regard to this definition, the following outcome variables were differentiated in the analyses: unrecommended sleep duration (defined as a duration of sleep that does not meet the recommended duration), sleep-onset difficulties, trouble sleeping, and daytime sleepiness. We assessed sleep duration according to the questions: “What time do you usually fall asleep during the week?” and “What time do you usually wake up or are woken up in the morning during the week?” To detect an insufficient sleep duration, answers were coded on the basis of the recommended sleep duration of the American Academy of Sleep Medicine [11]. Unrecommended sleep duration was defined as any duration of sleep that did not meet the recommended sleep duration and included sleeping longer than 10 or fewer than 8 h per night (“yes”) versus a reported sleep duration between 8 and 10 h per night (“no”). The variables sleep-onset difficulties, trouble sleeping, and daytime sleepiness were rated by adolescents on a three-point scale. Sleep-onset difficulties were assessed with the question: “How often do you fall asleep within 20 min?” Trouble sleeping was queried with: “How often do you wake up at night, when your parents think that you are asleep?” Daytime sleepiness was determined using the question: “How often do you feel sleepy during the day?” Participants were asked to choose between “usually (5–7 times/week)”, “sometimes (2–4 times/week)”, or “rarely (0–1 times/week)” For trouble sleeping and daytime sleepiness, the response categories were dichotomized as “yes” (usually/sometimes) and “no” (rarely). The response categories for the item “How often do you fall asleep within 20 min?” were dichotomized into “usually” and “sometimes/rarely”. Thereafter, the item was reversed as sleep-onset difficulties (“yes” versus “no”). 

The independent variable PA was assessed with one question: “How many days in a normal week are you active for at least 60 min per day?” Answers were given on a scale from zero to seven. In line with the recommendation from the WHO [24], sufficient PA was defined as being physically active for a minimum of 60 min on 7 days each week (“yes” versus “no”). 

The moderator variable SES is a score calculated using the net equivalent income of the household, educational, and occupational status of the parents. The categories “low”, “middle”, and “high” SES were calculated for the current study population as the lowest 20%, middle 60%, and highest 20% of participants [38]. 

Screen time, personal resources, and emotional and behavioral problems, as well as age, were included in all regression models as control variables. Normal screen time was defined as no more than 2 h of screen time per day [39]. For adolescents’ personal resources, a four-point scale instrument [40] was used. Personal resources were asked using a 5-item scale developed specially for the KiGGS study [40,41]. The scale includes items from the self-efficacy scale of Schwarzer and Jerusalem [42], the optimism scale of the Bern questionnaire on wellbeing [43], and the Sense of Coherence Scale [44]. Emotional and behavioral problems among adolescents were measured using the Strengths and Difficulties Questionnaire (SDQ) with the categories “conspicuous”, “borderline”, and “not conspicuous” [45,46]. Adolescents’ age was included as a categorical variable.

### 2.3. Statistical Analyses 

In the first step, we calculated the prevalence of sleep difficulties in total as well as stratified by PA and by SES in adolescent boys and girls. Bivariate associations are tested for independence using the Rao–Scott corrected chi-square statistic, which is transformed into an F statistic with noninteger degrees of freedom to account for the complex survey design [47,48].

In the second step, multivariate analyses were conducted. In Model 1, logistic regression analyses were calculated with sleep variables as the dependent and PA as the independent variable. In Model 2, logistic regression analyses were conducted with sleep variables as the dependent variable and with the interaction of PA and SES. Additionally, predicted margins were calculated and used for the visualization (the odds ratios for the logistic regressions with interaction terms are presented in the appendix; see Table A2). All results were stratified for girls and boys. Models 1 and 2 were adjusted for age, screen time, emotional and behavioral problems, and personal resources. To test whether the interaction between PA and SES is significant, the Wald test was used. Test results with an alpha level below 0.05 were reported as significant.

All analyses were carried out with weighted variables using the “svy” command for survey data in Stata/SE 15.1 (StataCorp LLC, College Station, TX, USA). 

**Figure 1:**
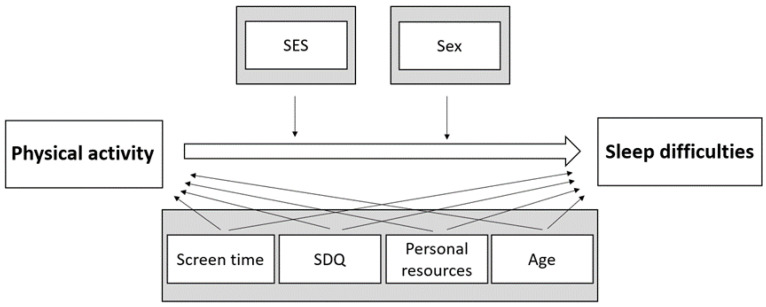
Directed acyclic graph underlying the analyses of the association between physical activity and sleep difficulties. SES, socioeconomic status; SDQ, Strengths and Difficulties Questionnaire.

Figure 1 depicts the underlying directed acyclic graph for the analyses, which displays the relationships assumed in the analyses.

## 3. Results

Table 1 presents a description of the study sample. Focusing on PA, 11.4% of adolescent girls and 18.2% of adolescent boys reported being physically active for a minimum of 60 min every day. The prevalence of sleep difficulties in total as well as stratified by PA in adolescent boys and girls can be found in Table 2. The prevalence of sleep difficulties stratified by SES is presented in Table A1 in the appendix.

### 3.1. Unrecommended Sleep Duration

**Figure 2:**
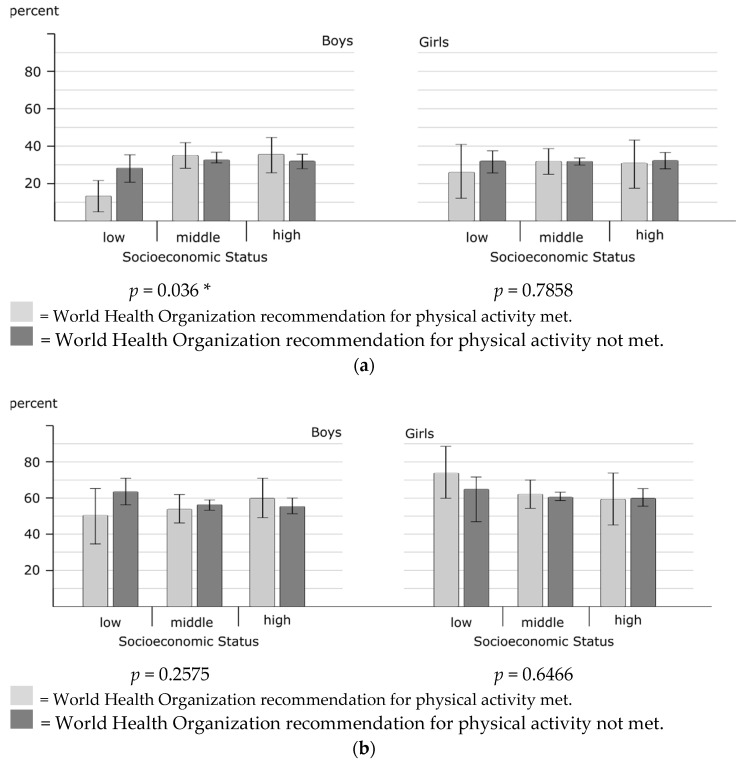

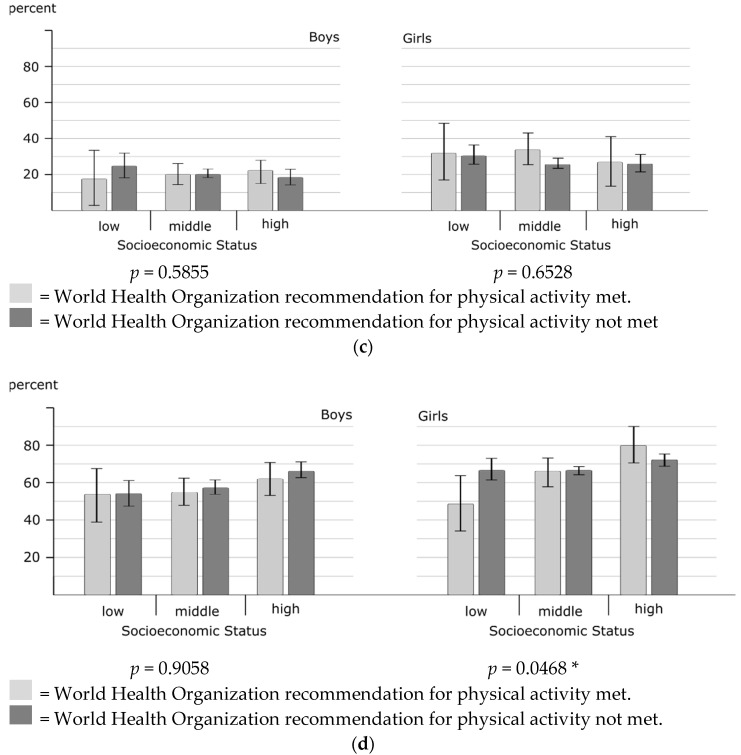
Predicted probabilities for sleep difficulties according to meeting the recommended physical activity level and socioeconomic status, stratified by sex. Results of logistic regression models with interaction terms of physical activity and socioeconomic status: adjusted for age, screen time, emotional and behavioral problems, and personal resources. *p* values calculated with the Wald-test; * *p* < 0.05. (**a**) Unrecommended sleep duration; (**b**) Sleep-onset difficulties; (**c**) Trouble sleeping; (**d**) Daytime sleepiness.

A total of 32.0% of male adolescents and 31.7% of female adolescents did not meet the sleep duration recommendation, with no differences between girls and boys (see Table 2). For girls, having an unrecommended sleep duration was significantly associated with not being physically active (see Table 2); however, this was not the case for boys. After adjusting for age, screen time, personal resources, and emotional and behavioral problems (Model 1), the association between sleep duration and PA was not significant in girls or boys (see Table 3). For boys, SES significantly moderates the relationship between PA and sleep duration (*p* = 0.037). Figure 2 shows that boys in the low-SES group had a greater probability of not meeting the recommended sleep duration when they did not meet the recommendation for PA. Such differences were not seen for boys in the middle- or high-SES groups (Figure 2a). In girls, we found no significant moderating effect of SES on the relationship between PA and sleep duration (*p* = 0.786) (Figure 2a).

### 3.2. Sleep-Onset Difficulties

In our sample, 57.3% of the male adolescents and 61.7% of female adolescents reported having sleep-onset difficulties (see Table 2). Sleep-onset difficulties were not associated with meeting the recommendation for PA in either boys or girls (see Table 2) and remained nonsignificant after adjusting for several variables (see Table 3). Moreover, no significant moderating effect of SES was observed for boys (*p* = 0.258) nor girls (*p* = 0.647) (Figure 2b). 

### 3.3. Trouble Sleeping

Sex-stratified results showed that 20.7% of adolescent boys and 27.6% of adolescent girls reported trouble sleeping (see Table 2). Trouble sleeping was not associated with meeting the recommendation for PA in boys or girls (see Table 2 and Table 3). Moreover, no significant interaction between PA and SES was found for boys (*p* = 0.586) nor girls (*p* = 0.653) (Figure 2c).

### 3.4. Daytime Sleepiness

Having daytime sleepiness at least twice a week was reported by 88.1% of adolescent boys and 67.0% of adolescent girls (see Table 2). This was, therefore, the most commonly reported sleep difficulty. For girls, daytime sleepiness was associated with not meeting the recommendation for PA (see Table 2). In the multivariate models with controlling for several variables, no significant association of PA with daytime sleepiness was found in either girls or boys (see Table 3). With regard to daytime sleepiness, the interaction of SES and PA was significant for girls (*p* = 0.047). Figure 2d shows that, if PA recommendation were met, girls in the low-SES group were less likely to report daytime sleepiness than girls in the high-SES group. No moderating effect of SES on the association between PA and daytime sleepiness was seen for boys (*p* = 0.906) (see Figure 2d).

Detailed results of the regression models for interaction of PA with SES for adolescent boys and girls can be found in the appendix (see Table A2).

## 4. Discussion

In the present study, we examined the prevalence of different sleep difficulties (unrecommended sleep duration, sleep-onset difficulties, trouble sleeping, and daytime sleepiness) among adolescent girls and boys in Germany, the association of sleep difficulties with PA according to WHO recommendations, and the moderating effect of SES on this association.

With regard to the first research question, we found a high number of adolescents with sleep difficulties. Most adolescents reported daytime sleepiness, although sleep-onset difficulties were also frequently mentioned. Fewer but still a large proportion of adolescents reported a sleep duration that did not meet the recommendation as well as trouble sleeping. An important finding is that diverse sleep variables yielded differing results. We agree with other studies [5,6,7,8], which concluded that it is useful to include different sleep variables in the analyses, because the complexity of sleep cannot be reflected by only one sleep variable.

Focusing on meeting the recommendation of sleep, around 32% of the girls and boys in this study reported an unrecommended sleep duration. We followed the American Academy of Sleep Medicine, which includes a range for a good sleep duration [11]. It is not recommended to sleep longer or shorter than this recommendation. It is recommended for adolescents to sleep between 8 and 10 h per night. Other studies defined a sufficient sleep duration as 8 h of sleep or more. Using this definition, studies have revealed that between 34.5% and 52.2% of adolescents do not meet the sleep duration recommendation [12,13,14]. Surprisingly, even though the definition in this analysis includes fewer hours and excludes excessive hours of sleep, more adolescents met the sleep duration recommendation in comparison with other studies. 

In terms of sleep-onset difficulties, the HBSC study in Europe found that approximately 20% of children and adolescents reported these difficulties. However, the prevalence of sleep-onset difficulties varied among countries between 9.6% of adolescents in the Ukraine to 37.4% in France. In Germany, 13.4% of adolescents reported sleep-onset difficulties in 2002 and 19.9% in 2014 [15]. The prevalence found in this analysis was much higher than in other studies. This may be owing to the fact that sleep-onset difficulty was measured slightly differently in the HBSC study than in the KiGGS study, with the KiGGS survey setting a specific time of 20 min for falling asleep and the HBSC study querying more broadly about perceived difficulties in falling asleep in the previous 6 months.

In terms of trouble sleeping, a study from Finland found that 30.4% of 3- to 6-year-old children had sleeping problems during the night [16]. Nevertheless, any comparison should be made with caution because of the different ages of the study participants. No studies were found regarding trouble sleeping in adolescents, which makes it difficult to rate the results. 

The present prevalence of daytime sleepiness was in line with the daytime sleepiness prevalence of 65.1% among adolescents found in an Indian study [49], whereas in Norway, only 18% of adolescents reported daytime sleepiness [5]. These large differences in prevalence may be explained by country differences. However, other aspects may also explain these variations, such as media use or family characteristics. It is important to understand the underlying mechanisms to mitigate adolescent daytime sleepiness. Similar to trouble sleeping, studies regarding daytime sleepiness are lacking. A Spanish study focusing on morning tiredness showed similar prevalence as the present study for daytime sleepiness. Morning tiredness was reported by 67.1% of the boys and 72.7% of the girls surveyed [8], which might enable comparisons of daytime sleepiness with morning tiredness. However, such comparisons should be made with caution. A comparison of prevalence reveals large differences between studies. 

In our study, we found significant associations in girls between PA and the variable sleep duration not meeting the recommendations as well as PA and daytime sleepiness (research question 2). Another study also found differences between girls and boys. In one study, the association between PA and sufficient sleep was significant for girls only [12]. The results of another study partly support these findings. The authors reported small differences for PA and sleep disturbances between girls and boys; however, these differences were not significant [50]. Controlling for several variables in our model resulted in no significant associations between PA and the sleep variables. Xu et al. [12] found that screen time was important for some sleep variables in combination with PA [12]. Because we included screen time and the other control variables at the same time, we cannot quantify the mediating effect of screen time alone on the association between PA and sleep. 

To answer our research question 3, we analyzed the association of PA and sleep difficulties after adjusting for age, screen time, and personal resources as well as emotional and behavioral problems. Mixed results regarding the relationship of PA and sleep can be found in the literature. In the present study, after introducing the variables into the model, we did not find a significant impact of PA on sleep difficulties in either boys or girls. This finding is in line with those of other studies analyzing sleep duration [25,51] and sleep-onset difficulties [52]. Similar to findings of the HBSC study [15], we found no significant association between PA and sleep-onset difficulties. It should be noted that the findings of that past study were based on data from all countries combined. The analyses showed that the strength of the association between PA and sleep-onset difficulties varied among European countries [15]. However, other studies have found associations between sleep variables and PA [10,12,22]. Xu et al. [12] showed that students who meet the recommendations for PA had 50% lower odds of reporting sufficient sleep duration than those who did not meet the PA recommendations. However, for other variables of sleep problems, such as trouble falling asleep or not feeling rested, no significant associations were observed in that study [12]. A meta-analysis showed significant associations between subjective PA and objective sleep [10]. A causal relationship between sleep and PA was also identified because adolescents reported better sleep quality [22]. However, in that study, no significant differences were found for PA and sleepiness and sleep duration after exercising for 3 weeks [22]. When comparing the findings of this analyses with those of other studies, it should be noted that sleep measurements differ highly across studies [10,19]. This makes it difficult to compare the results. 

This was the first study to focus on PA and sleep difficulties and the moderating effect of SES (research question 4). Our main finding is that SES was found to moderate some but not all relationships between PA and sleep difficulties. Without considering SES, differences in the relationship of PA with some sleep difficulties according to SES would not be evident. The analyses of sleep duration and PA in boys and daytime sleepiness and PA in girls showed a significant moderation according to SES. Girls and boys in the low SES-group had lower odds of experiencing sleep difficulties compared with adolescents in other SES groups when meeting the PA recommendation. Another study found a moderating effect of SES on the association between adolescents sleep and socioemotional and cognitive difficulties [53]. The authors concluded that adolescents reporting better sleep quality and longer sleep hours reported less difficulties. This is a highly interesting finding, because public health interventions face a prevention dilemma, as it is often presumed that people with low SES, in particular, do not benefit from prevention programs [54]. The results by El-Sheikh et al. [53] also conclude that adolescents with low SES benefit highly from good sleep quality and quantity. The present findings indicated that the sleep of adolescents with low SES in particular would benefit from PA promotion programs. 

As a synthesis of the results of the present study, it can be stated that a high proportion of adolescents obtain poor-quality sleep. As other studies have shown, sleep difficulties are important for adolescents’ health and development [1,2,3,4,16,53,55,56]. This particularly affects adolescents from families with low SES [53,55,56]. It is therefore important to identify protective factors in order to improve the sleep of adolescents with low SES, in particular. Contrary to our assumption, PA is only marginally associated with the sleep difficulties measured here. However, the sleep of adolescents with low SES, in particular, seemed to benefit from physical activity. 

This analysis has several limitations. In the present study, PA and sleep duration were categorized in line with the recommendations. These recommendations can be interpreted critically. A narrative review concluded that each person has slightly different sleep needs, which result in different sleep patterns [57]. These patterns exist owing to a variety of different genetic, behavioral, environmental, and social factors. Sleep recommendations give an overview of sleep durations that provide the most favorable health benefits, although there is no claim that these sleep durations are ideal for everyone. The best sleep duration for each person needs to be decided individually [57]. Other authors have stated that the recommendations for sufficient sleep should be reviewed and should include age, gender, chronotype, and cohort, as well as cultural, environmental, and other possible factors [4]. 

It should be noted that sleep measurements differ greatly across studies [10,19]. Currently, there is no optimal method to measure sleep [10], making it difficult to compare studies and results.

In this study, sufficient PA was defined using the WHO PA recommendation [24]. However, other ways of measuring PA would also be important to consider, such as the intensity of PA or the time of day when PA was conducted. 

In the present study, the analyses were adjusted for screen time, emotional and behavioral problems, personal resources, and age. Including all these variables might have resulted in overadjustment. 

Additionally, the present analyses were conducted using cross-sectional data collected for the purpose of providing a broad overview of different aspects of child and adolescent health in Germany. Therefore, the data can show associations but no causal effect. For those effects, longitudinal data would be necessary. Furthermore, because our study was not conducted to investigate sleep in detail, some important impact factors, such as background noise while sleeping or sleep hygiene, are missing. Sleep hygiene would be interesting to include in further analyses, because it seems to be important for sleeping well and for good sleep quality, as well as for positive impacts on mental and physical health [58]. Sleep hygiene involves a variety of different practices and habits during the day and right before going to bed. Those practices and habits include limiting daytime naps to 30 min, avoiding stimulants such as caffeine before bedtime, engaging in exercise for good sleep quality, maintaining a regular bedtime routine, having a supportive sleep environment, and more [58].

The number of adolescents who met the recommended amount of PA in this study was relatively small, which resulted in wide confidence intervals. A higher prevalence of daily PA would provide more reliable results.

All information used in this study was provided subjectively by adolescents. This might limit the data; however, a study by Kalak et al. [59] showed that objectively and subjectively measured sleep had similar results regarding psychological well-being. Another study reported that subjectively reported sleep duration was overestimated by 48 min compared with objectively measured sleep duration in an adult sample. Objective and subjective reports have a moderate correlation [60], although it should be kept in mind that this evidence was among a study population of adults. This aspect is supported by another study reporting that subjective PA is more important than objective PA in analyzing the association with sleep [21]. 

One strength of our analyses is the inclusion of different variables analyzing sleep difficulties among adolescents, as recommended in other studies [5,6,7,8]. A further strength is the large representative sample in the cross-sectional KiGGS study Wave 2, which made it possible to calculate valid prevalence of sleep difficulties in Germany and to stratify those factors by PA, SES, and sex concurrently. Furthermore, this was the first study to include SES as a moderating variable in the relationship between PA and different aspects of sleep difficulties.

## 5. Conclusions

In Germany, a large proportion of adolescent girls and boys have sleep difficulties. Therefore, improving sleep in adolescents should be a target of health promotion. Our findings revealed that PA potentially affects adolescents’ sleep differently in dependence on the SES of the family. Thus, future sleep promotion programs should consider differences according to the sex, SES, and PA of adolescents. The present results are especially interesting for adolescents with low SES, as this population would likely benefit most from preventive interventions, which could help to reduce health inequalities in adolescents. 

For the purpose of deriving recommendations for sleep promotion strategies, future research should include information on sleep hygiene to more accurately illustrate the complexity of the topic under consideration. Longitudinal studies could be helpful in analyzing causal pathways, as should intervention studies to evaluate programs on improving sleep in youth. Additionally, the impact of PA, as well as screen time, on sleep quality in adolescents should be examined in greater detail. This aspect is particularly important during the COVID-19 pandemic, when sporting activities have been severely restricted and media use has increased to a high level among young people in Germany [61].

## Figures and Tables

**Table 1:** Description of study sample by sex.

Sleep Variables		Total	Total	Boys	Boys	Girls	Girls
		*n* Unweighted	% Weighted	*n* Unweighted	% Weighted	*n* Unweighted	% Weighted
		6599		3176		3423	
Unrecommended sleep duration	Yes	1789	31.9	830	32.1	959	31.7
	No	4348	68.1	2097	67.9	2251	68.3
Sleep-onset difficulties	Yes	3603	59.3	1628	57.2	1975	61.7
	No	2467	40.7	1269	42.8	1198	38.3
Trouble sleeping	Yes	1431	24.0	584	20.8	847	27.5
	No	4655	76.0	2317	79.3	2338	72.5
Daytime sleepiness	Yes	3843	62.4	1706	58.2	2137	67.0
	No	2251	37.6	1200	41.8	1051	33.1
PA recommendation	Yes	890	14.8	252	18.2	365	11.1
	No	5248	85.2	2403	81.8	2845	88.9
SES	Low	874	20.8	410	20.2	464	21.6
	Middle	4103	61.9	1967	61.7	2136	62.0
	High	1477	17.3	734	18.2	743	16.4
Age	11–13 years	3026	40.8	1496	40.6	1530	41.1
	14–17 years	3573	59.2	1680	59.4	1893	59.4
Screen time	Low	1470	23.1	561	18.8	909	27.7
	High	4685	76.9	2376	81.3	2309	72.32
Emotional and behavioral problems	Inconspicuous	241	4.1	77	2.8	164	5.48
	Borderline	504	8.0	211	6.8	293	9.21
	Conspicuous	5400	87.9	2644	90.3	2756	85.32
Personal resources	High	5922	88.6	2924	91.4	2998	85.54
	Low	677	11.5	252	8.6	425	14.46

SES, socioeconomic status.

**Table 2:** Sleep difficulties in adolescent girls and boys stratified by physical activity (met recommendation).

Sleep Variables	Total	Physical Activity (Met Recommendation)		
		Yes	No	*p* Value
	% (95% CI)	% (95% CI)	% (95% CI)	
Boys				
Unrecommended sleep duration	32.0 (29.7–34.4)	29.0 (23.9–34.7)	32.7 (30.2–35.3)	0.2290
Sleep-onset difficulties	57.3 (54.7–59.8)	53.6 (47.5–59.5)	58.1 (55.5–60.6)	0.1388
Trouble sleeping	20.7 (18.7–23.0)	20.1 (16.0–24.9)	20.9 (18.7–23.3)	0.7469
Daytime sleepiness	58.1 (55.7–60.6)	54.1 (47.8–60.3)	59.0 (56.3–61.7)	0.1539
Girls				
Unrecommended sleep duration	31.7 (29.7–33.8)	22.4 (17.4–28.3)	32.9 (30.7–35.1)	0.0012 *
Sleep-onset difficulties	61.7 (59.4–64.0)	65.0 (57.8–71.6)	61.3 (58.8–63.7)	0.3350
Trouble sleeping	27.6 (25.6–29.7)	29.3 (22.9–36.6)	27.4 (25.2–29.6)	0.6040
Daytime sleepiness	67.0 (65.0–69.0)	56.2 (49.3–62.9)	68.4 (66.2–70.5)	0.0007 *

CI, confidence interval; *p* values calculated with the Rao–Scott corrected chi-square statistic; * *p* < 0.05.

**Table 3:** Relationship between sleep difficulties and PA in adolescent boys and girls ^1^.

		Unrecommended Sleep Duration		Sleep-Onset Difficulties		Trouble Sleeping		Daytime Sleepiness	
		OR (95% CI)	*p*	OR (95% CI)	*p*	OR (95% CI)	*p*	OR (95% CI)	*p*
Boys									
PA recommendation	No	Ref.	-	Ref.	-	Ref.	-	Ref.	-
	Yes	0.96 (0.70, 1.30)	0.784	0.86 (0.67, 1.09)	0.205	0.98 (0.73, 1.32)	0.893	0.91 (0.68, 1.21)	0.501
Girls									
PA recommendation	No	Ref.	-	Ref.	-	Ref.	-	Ref.	-
	Yes	0.89 (0.63, 1.26)	0.499	1.14 (0.83, 1.57)	0.401	1.26 (0.88, 1.81)	0.199	0.80 (0.57, 1.13)	0.211

^1^ Logistic regression models; all models adjusted for: screen time, emotional and behavioral problems, personal resources, age. PA, physical activity; OR, odds ratio; CI, confidence interval; *p* values calculated with the Wald-test.

## Data Availability

The data set cannot be made publicly available, because informed consent from study participants did not cover public deposition of data. However, the minimal data set underlying the findings is archived in the “Health Monitoring” Research Data Centre at the Robert Koch Institute (RKI) and can be accessed by all interested researchers on site. The “Health Monitoring” Research Data Centre is accredited by the German Data Forum according to uniform and transparent standards (http://www.ratswd.de/en/data-infrastructure/rdc). On-site access to the minimal data set is possible at the Secure Data Centre of the RKI’s “Health Monitoring” Research Data Centre, which is located at General-Pape-Straße 64 in Berlin, Germany. Requests should be submitted to Dr. Ronny Kuhnert at the Robert Koch Institute, “Health Monitoring” Research Data Centre, General-Pape-Straße 64, 12101 Berlin, Germany (e-mail: fdz@rki.de).

## Appendix A

**Table A1:** Sleep difficulties in adolescents stratified by socioeconomic status.

Sleep Variables	Socioeconomic Status				
	Total	SES Low	SES Middle	SES High	*p* Value
	% (95% CI)	% (95% CI)	% (95% CI)	% (95% CI)	
Boys					
Unrecommended sleep duration	32.1 (29.8–34.4)	28.1 (22.1–35.0)	33.9 (30.84–37.11)	30.17 (26.6–43.0)	0.1596
Sleep-onset difficulties	57.1 (54.5–59.7)	62.5 (56.0–68.6)	56.1 (53.0–59.1)	54.9 (50.6–59.0)	0.0689
Trouble sleeping	20.7 (18.6–23.0)	24.9 (18.7–32.2)	20.2 (17.7–23.0)	17.8 (14.5–21.8)	0.1612
Daytime sleepiness	57.8 (55.3–60.3)	55.0 (48.14–61.6)	57.6 (54.5–60.7)	61.6 (56.8–66.1)	0.2785
Girls					
Unrecommended sleep duration	31.8 (29.8–33.9)	34.7 (28.9–42.1)	31.7 (29.3–34.2)	28.5 (24.3–33.1)	0.2542
Sleep-onset difficulties	61.8 (59.5–64.1)	67.0 (60.7–72.7)	60.4 (57.5–63.3)	60.1 (55.4–64.7)	0.0890
Trouble sleeping	27.3 (25.2–29.5)	33.1 (27.6–39.1)	26.2 (23.8–28.9)	23.6 (19.7–28.1)	0.0146 *
Daytime sleepiness	66.8 (64.8–68.8)	67.8 (62.4–72.8)	66.1 (63.4–68.6)	68.5 (63.9–72.8)	0.6385

CI, confidence interval, SES, socioeconomic status; *p* values calculated with the Rao–Scott corrected chi-square statistic; * *p* < 0.05.

**Table A2:** Interaction between PA and SES stratified by sex (logistic regression models; all models adjusted for: screen time, emotional and behavioral problems, personal resources, age).

		Unrecommended Sleep Duration		Sleep-Onset Difficulties		Trouble Sleeping		Daytime Sleepiness	
		OR (95% CI)	*p*	OR (95% CI)	*p*	OR (95% CI)	*p*	OR (95% CI)	*p*
Boys									
PA recommendation	No	Ref.	-	Ref.	-	Ref.	-	Ref.	-
	Yes	0.34 (0.15, 0.80)	0.014	0.58 (0.28, 1.18)	0.131	0.64 (0.24, 1.70)	0.371	0.99 (0.48, 2.04)	0.969
SES	Low	Ref.	-	Ref.	-	Ref.	-	Ref.	-
	Middle	1.27 (0.80, 2.02)	0.312	0.74 (0.54, 1.01)	0.054	0.76 (0.50, 1.17)	0.210	1.15 (0.82, 1.61)	0.413
	High	1.23 (0.76, 1.98)	0.392	0.71 (0.49, 1.01)	0.058	0.68 (0.42, 1.10)	0.116	1.73 (0.17, 2.56)	0.006
PA#SES	Yes#Low	Ref.	-	Ref.	-	Ref.	-	Ref.	-
	Yes#Middle	3.32 (1.27, 8.66)	0.015	1.56 (0.71, 3.44)	0.270	1.56 (0.54, 4.51)	0.410	0.91 (0.41, 2.00)	0.815
	Yes#High	3.54 (1.26, 9.93)	0.017	2.10 (0.87, 5.07)	0.100	1.82 (0.58, 5.66)	0.300	0.83 (0.36, 1.95)	0.675
Girls									
PA recommendation	No	Ref.	-	Ref.	-	Ref.	-	Ref.	-
	Yes	0.71 (0.29, 1.74)	0.455	1.54 (0.70, 3.39)	0.285	1.08 (0.49, 2.36)	0.848	0.42 (0.19, 0.95)	0.036
SES	Low	Ref.	-	Ref.	-	Ref.	-	Ref.	-
	Middle	0.99 (0.70, 1.40)	0.938	0.82 (0.59, 1.15)	0.248	0.78 (0.57, 1.07)	0.123	1.00 (0.71, 1.41)	0.989
	High	1.02 (0.66, 1.56)	0.937	0.81 (0.56, 1.16)	0.241	0.79 (0.54, 1.17)	0.240	1.36 (0.94, 1.96)	0.099
PA#SES	Yes#Low	Ref.	-	Ref.	-	Ref.	-	Ref.	-
	Yes#Middle	1.41 (0.52, 3.83)	0.502	0.70 (0.30, 1.64)	0.407	1.39 (0.57, 3.40)	0.462	2.31 (0.92, 5.79)	0.074
	Yes#High	1.30 (0.51, 3.32)	0.586	0.63 (0.24, 1.71)	0.365	0.98 (0.31, 3.07)	0.975	3.80 (1.32, 10.93)	0.014

CI, confidence interval, SES, socioeconomic status; *p* values calculated with the Rao–Scott corrected chi-square statistic, # = interaction.

## References

[1] Colrain I.M., Baker F.C. (2011). Changes in sleep as a function of adolescent development. Neuropsychol. Rev..

[2] Brand S., Lemola S., Mikoteit T., Holsboer-Trachsler E., Kalak N., Bahmani D.S., Puhse U., Ludyga S., Gerber M. (2019). Schlaf und Befindlichkeit bei Kindern und Jugendlichen—Ein narratives Review. [Sleep and well-being in children and adolescents—A narrative review]. Prax. Der Kinderpsychol. Und Kinderpsychiatr..

[3] Chaput J.P., Gray C.E., Poitras V.J., Carson V., Gruber R., Olds T., Weiss S.K., Connor Gorber S., Kho M.E., Sampson M. (2016). Systematic review of the relationships between sleep duration and health indicators in school-aged children and youth. Appl. Physiol. Nutr. Metab..

[4] Shochat T., Cohen-Zion M., Tzischinsky O. (2014). Functional consequences of inadequate sleep in adolescents: A systematic review. Sleep Med. Rev..

[5] Hestetun I., Svendsen M.V., Oellingrath I.M. (2018). Sleep problems and mental health among young Norwegian adolescents. Nord. J. Psychiatry.

[6] El-Sheikh M., Tu K.M., Erath S.A., Buckhalt J.A. (2014). Family stress and adolescents’ cognitive functioning: Sleep as a protective factor. J. Fam. Psychol..

[7] Dewald J.F., Meijer A.M., Oort F.J., Kerkhof G.A., Bogels S.M. (2010). The influence of sleep quality, sleep duration and sleepiness on school performance in children and adolescents: A meta-analytic review. Sleep Med. Rev..

[8] Segura-Jiménez V., Carbonell-Baeza A., Keating X.D., Ruiz J.R., Castro-Piñero J. (2015). Association of sleep patterns with psychological positive health and health complaints in children and adolescents. Qual. Life Res..

[9] Taylor D.J., Bramoweth A.D. (2010). Patterns and Consequences of Inadequate Sleep in College Students: Substance Use and Motor Vehicle Accidents. J. Adolesc. Health.

[10] Lang C., Kalak N., Brand S., Holsboer-Trachsler E., Puhse U., Gerber M. (2016). The relationship between physical activity and sleep from mid adolescence to early adulthood. A systematic review of methodological approaches and meta-analysis. Sleep Med. Rev..

[11] Paruthi S., Brooks L.J., D’Ambrosio C., Hall W.A., Kotagal S., Lloyd R.M., Malow B.A., Maski K., Nichols C., Quan S.F. (2016). Recommended Amount of Sleep for Pediatric Populations: A Consensus Statement of the American Academy of Sleep Medicine. J. Clin. Sleep Med..

[12] Xu F., Adams S.K., Cohen S.A., Earp J.E., Greaney M.L. (2019). Relationship between Physical Activity, Screen Time, and Sleep Quantity and Quality in US Adolescents Aged 16-19. Int. J. Environ. Res. Public Health.

[13] Kosticova M., Geckova A.M., Dobiasova E., Veselska Z.D. (2019). Insufficient sleep duration is associated with worse self-rated health and more psychosomatic health complaints in adolescents. Bratisl. Lek. Listy.

[14] Gireesh A., Das S., Viner R.M. (2018). Impact of health behaviours and deprivation on well-being in a national sample of English young people. BMJ Paediatr. Open.

[15] Ghekiere A., Van Cauwenberg J., Vandendriessche A., Inchley J., Gaspar de Matos M., Borraccino A., Gobina I., Tynjala J., Deforche B., De Clercq B. (2019). Trends in sleeping difficulties among European adolescents: Are these associated with physical inactivity and excessive screen time?. Int. J. Public Health.

[16] Simola P., Liukkonen K., Pitkaranta A., Pirinen T., Aronen E.T. (2014). Psychosocial and somatic outcomes of sleep problems in children: A 4-year follow-up study. Child Carehealth Dev..

[17] Poulain T., Vogel M., Sobek C., Hilbert A., Korner A., Kiess W. (2019). Associations Between Socio-Economic Status and Child Health: Findings of a Large German Cohort Study. Int. J. Environ. Res. Public Health.

[18] Chennaoui M., Arnal P.J., Sauvet F., Leger D. (2015). Sleep and exercise: A reciprocal issue?. Sleep Med. Rev..

[19] Fatima Y., Doi S.A., Mamun A.A. (2015). Longitudinal impact of sleep on overweight and obesity in children and adolescents: A systematic review and bias-adjusted meta-analysis. Obes. Rev. Off. J. Int. Assoc. Study Obes..

[20] Jakobsson M., Josefsson K., Hogberg K. (2019). Reasons for sleeping difficulties as perceived by adolescents: A content analysis. Scand. J. Caring Sci..

[21] Lang C., Brand S., Feldmeth A.K., Holsboer-Trachsler E., Puhse U., Gerber M. (2013). Increased self-reported and objectively assessed physical activity predict sleep quality among adolescents. Physiol. Behav..

[22] Kalak N., Gerber M., Kirov R., Mikoteit T., Yordanova J., Puhse U., Holsboer-Trachsler E., Brand S. (2012). Daily morning running for 3 weeks improved sleep and psychological functioning in healthy adolescents compared with controls. J. Adolesc. Health Off. Publ. Soc. Adolesc. Med..

[23] Caspersen C.J., Powell K.E., Christenson G.M. (1985). Physical activity, exercise, and physical fitness: Definitions and distinctions for health-related research. Public Health Rep..

[24] World Health Organization Global Recommendations on Physical Activity for Health. https://apps.who.int/iris/bitstream/handle/10665/44399/9789241599979_eng.pdf?sequence=1&isAllowed=y.

[25] Dalene K.E., Anderssen S.A., Andersen L.B., Steene-Johannessen J., Ekelund U., Hansen B.H., Kolle E. (2018). Cross-sectional and prospective associations between sleep, screen time, active school travel, sports/exercise participation and physical activity in children and adolescents. BMC Public Health.

[26] Juneau C.E., Benmarhnia T., Poulin A.A., Côté S., Potvin L. (2015). Socioeconomic position during childhood and physical activity during adulthood: A systematic review. Int. J. Public Health.

[27] Elhakeem A., Cooper R., Bann D., Hardy R. (2015). Childhood socioeconomic position and adult leisure-time physical activity: A systematic review. Int. J. Behav. Nutr. Phys. Act..

[28] Stalsberg R., Pedersen A.V. (2010). Effects of socioeconomic status on the physical activity in adolescents: A systematic review of the evidence. Scand. J. Med. Sci. Sports.

[29] World Health Organization Adolescent Obesity and Related Behaviours: Trends and Inequalities in the WHO European Region, 2002–2014. https://www.euro.who.int/__data/assets/pdf_file/0019/339211/WHO_ObesityReport_2017_v3.pdf.

[30] Kim J., Noh J.W., Kim A., Kwon Y.D. (2020). Demographic and Socioeconomic Influences on Sleep Patterns among Adolescent Students. Int. J. Environ. Res. Public Health.

[31] Felden É.P., Leite C.R., Rebelatto C.F., Andrade R.D., Beltrame T.S. (2015). Sleep in adolescents of different socioeconomic status: A systematic review. Rev. Paul. De Pediatr. Orgao Of. Da Soc. De Pediatr. De Sao Paulo.

[32] Tonetti L., Fabbri M., Natale V. (2008). Sex difference in sleep-time preference and sleep need: A cross-sectional survey among Italian pre-adolescents, adolescents, and adults. Chronobiol. Int..

[33] Miguez M.J., Bueno D., Perez C. (2020). Disparities in Sleep Health among Adolescents: The Role of Sex, Age, and Migration. Sleep Disord..

[34] James S., Chang A.M., Buxton O.M., Hale L. (2020). Disparities in adolescent sleep health by sex and ethnoracial group. SSM Popul. Health.

[35] Lemola S., Räikkönen K., Scheier M.F., Matthews K.A., Pesonen A.K., Heinonen K., Lahti J., Komsi N., Paavonen J.E., Kajantie E. (2011). Sleep quantity, quality and optimism in children. J. Sleep Res..

[36] Schlarb A.A., Kulessa D., Gulewitsch M.D. (2012). Sleep characteristics, sleep problems, and associations of self-efficacy among German university students. Nat. Sci. Sleep.

[37] Mauz E., Gößwald A., Kamtsiuris P., Hoffmann R., Lange M., Schenck U.v., Allen J., Butschalowsky H., Frank L., Hölling H. (2017). Neue Daten für Taten. Die Datenerhebung zur KiGGS Welle 2 ist beendet. [New data for action. The data collection for KiGGS Wave 2 is completed]. J. Health Monit..

[38] Lampert T., Hoebel J., Kuntz B., Müters S., Kroll L.E. (2018). Messung des sozioökonomischen Status und des subjektiven sozialen Status in KiGGS Welle 2. [Measurement of socioeconomic status and subjective social status in KiGGS wave 2]. J. Health Monit..

[39] Manz K., Schlack R., Poethko-Müller C., Mensink G., Finger J., Lampert T. (2014). Körperlich-sportliche Aktivität und Nutzung elektronischer Medien im Kindes- und Jugendalter. [Physical activity and use of electronic media in childhood and adolescence]. Bundesgesundheitsblatt Gesundh. Gesundh..

[40] Bettge S., Ravens-Sieberer U. (2003). Schutzfaktoren für die psychische Gesundheit von Kindern und Jugendlichen—Empirische Ergebnisse zur Validierung eines Konzepts [Protective factors for mental health of children and adolescents--empirical results validating a concept]. Gesundheitswesen.

[41] Erhart M., Hölling H., Bettge S., Ravens-Sieberer U., Schlack R. (2007). Der Kinder- und Jugendgesundheitssurvey (KiGGS): Risiken und Ressourcen für die psychische Entwicklung von Kindern und Jugendlichen. [The Child and Adolescent Health Survey (KiGGS): Risks and Resources for the Mental Development of Children and Adolescents]. Bundesgesundheitsblatt-Gesundh.-Gesundh..

[42] Schwarzer R., Jerusalem M. (1999). Skalen zur Erfassung von Lehrer- und Schülermerkmalen. Dokumentation der Psychometrischen Verfahren im Rahmen der Wissenschaftlichen Begleitung des Modellversuchs Selbstwirksame Schulen [Scales for the Assessment of Teacher and Student Characteristics. Documentation of the Psychometric Procedures within the Framework of the Scientific Monitoring of the Pilot Project Self-Efficacious Schools].

[43] Grob A., Lüthi R., Kaiser F.G., Flammer A. (1991). Berner Fragebogen zum Wohlbefinden Jugendlicher (BFW). [The Bern Subjective Well-Being Questionnaire for Adolescents (BFW)]. Diagnostica.

[44] Kern R., Rasky E., Noack R.H. (1995). Indikatoren für Gesundheitsförderung in der Volksschule.

[45] Goodman R., Ford T., Simmons H., Gatward R., Meltzer H. (2000). Using the Strengths and Difficulties Questionnaire (SDQ) to screen for child psychiatric disorders in a community sample. Br. J. Psychiatry.

[46] Klipker K., Baumgarten F., Göbel K., Lampert T., Hölling H. (2018). Psychische Auffälligkeiten bei Kindern und Jugendlichen in Deutschland—Querschnittergebnisse aus KiGGS Welle 2 und Trends. [Mental disorders in children and adolescents in Germany-cross-sectional results from KiGGS wave 2 and trends]. J. Health Monit..

[47] Rao J.N.K., Scott A.J. (1981). The Analysis of Categorical Data from Complex Sample Surveys: Chi-Squared Tests for Goodness of Fit and Independence in Two-Way Tables. J. Am. Stat. Assoc..

[48] Rao J.N.K., Scott A.J. (1984). On Chi-Squared Tests for Multiway Contingency Tables with Cell Proportions Estimated from Survey Data. Ann. Stat..

[49] Murugesan G., Karthigeyan L., Selvagandhi P., Gopichandran V. (2018). Sleep patterns, hygiene and daytime sleepiness among adolescent school-goers in three districts of Tamil Nadu: A descriptive study. Natl. Med. J. India.

[50] Brand S., Kalak N., Gerber M., Clough P.J., Lemola S., Sadeghi Bahmani D., Pühse U., Holsboer-Trachsler E. (2017). During early to mid adolescence, moderate to vigorous physical activity is associated with restoring sleep, psychological functioning, mental toughness and male gender. J. Sports Sci..

[51] Ortega F.B., Ruiz J.R., Labayen I., Kwak L., Harro J., Oja L., Veidebaum T., Sjöström M. (2011). Sleep duration and activity levels in Estonian and Swedish children and adolescents. Eur. J. Appl. Physiol..

[52] Marques A., Demetriou Y., Tesler R., Gouveia E.R., Peralta M., Matos M.G. (2019). Healthy Lifestyle in Children and Adolescents and Its Association with Subjective Health Complaints: Findings from 37 Countries and Regions from the HBSC Study. Int. J. Environ. Res. Public Health.

[53] El-Sheikh M., Shimizu M., Philbrook L.E., Erath S.A., Buckhalt J.A. (2020). Sleep and development in adolescence in the context of socioeconomic disadvantage. J. Adolesc..

[54] Franskowiak P. Präventionsparadox. [Prevention Paradox]. https://leitbegriffe.bzga.de/alphabetisches-verzeichnis/praeventionsparadox/.

[55] Buzek T., Poulain T., Vogel M., Engel C., Bussler S., Körner A., Hiemisch A., Kiess W. (2019). Relations between sleep duration with overweight and academic stress-just a matter of the socioeconomic status?. Sleep Health.

[56] El-Sheikh M., Kelly R.J., Buckhalt J.A., Benjamin Hinnant J. (2010). Children’s sleep and adjustment over time: The role of socioeconomic context. Child Dev..

[57] Chaput J.P., Dutil C., Sampasa-Kanyinga H. (2018). Sleeping hours: What is the ideal number and how does age impact this?. Nat. Sci. Sleep.

[58] Sleep Foundation Sleep Hygiene. https://www.sleepfoundation.org/articles/sleep-hygiene.

[59] Kalak N., Gerber M., Kirov R., Mikoteit T., Puhse U., Holsboer-Trachsler E., Brand S. (2012). The relation of objective sleep patterns, depressive symptoms, and sleep disturbances in adolescent children and their parents: A sleep-EEG study with 47 families. J. Psychiatr. Res..

[60] Lauderdale D.S., Knutson K.L., Yan L.L., Liu K., Rathouz P.J. (2008). Self-reported and measured sleep duration: How similar are they?. Epidemiology.

[61] Schmidt S.C.E., Anedda B., Burchartz A., Eichsteller A., Kolb S., Nigg C., Niessner C., Oriwol D., Worth A., Woll A. (2020). Physical activity and screen time of children and adolescents before and during the COVID-19 lockdown in Germany: A natural experiment. Sci. Rep..

